# Complimentary Dinner to Dr. John B. Chapin

**Published:** 1904-10

**Authors:** 


### CORRESPONDENCE.

### Complimentary Dinner to Dr. John B. Chapin.

*Editor Buffalo Medical Journal:*

SIR—During the present year Dr. John B. Chapin, superintendent and physician of the Pennsylvania Hospital for the Insane, Philadelphia, will have completed a half-century's work in behalf of the insane. He was appointed assistant physician at the State Lunatic Asylum, Utica, New York, 1854. Afterward he became connected with the late Dr. George Cook in the conduct of Brigham Hall, Canandaigua, New York, and then, first as one of the building commissioners, and subsequently as physician and superintendent, with the Willard Asylum for the Chronic Insane, an institution made famous under his organisation and work.

Since 1884, he has been the medical chief of the Pennsylvania Hospital for the Insane, succeeding the late Dr. Kirkbride. His friends deem the completion of this long period of work, rounding out a half century in one department of medicine, a proper occasion on which to express to Dr. Chapin their personal affection and their appreciation of the extent and value of his work.

It is proposed to give Dr. Chapin a dinner some time in November or possibly December next, and to have his portrait painted for presentation to him and his family in honor of the event. To accomplish this purpose a committee has been formed to solicit subscriptions and to make all other arrangements. The committee is sure that you will consider it a privilege and honor to contribute to this purpose by publishing this statement.

Subscriptions may be sent by check or money order payable to

EDWARD N. BRUSH,
Sheppard and Enoch Pratt Hospital,
September 5, 1904. Station A, Baltimore, Md.

The following-named constitute the committee: Henry E. Allison, Matteawan State Hospital, Fishkill-on-Hudson, N. Y.; J. W. Babcock, State Hospital, Columbia, S. C.; Benjamin Blackford, Western State Hospital, Staunton, Va.; G. Alder Blumer, Butler Hospital, Providence, R. I.; Edward N. Brush, Sheppard and Enoch Pratt Hospital, Towson, Maryland; Dwight R. Burrell, Brigham Hall, Canandaigua, N. Y.; Edward Cowles, 419 Boylston street, Boston, Mass.; Richard Dewey, Milwaukee Sanitarium, Wauwautosa, Wis.; W. A. Gordon, Northern Hospital, Winnebago, Wis.; Henry M. Hurd, Johns Hopkins Hospital, Baltimore, Md.; A. E. Macdonald, Manhattan State Hospital, New York; S. Weir Mitchell, 1524 Walnut street, Philadelphia, Pa.; A. R. Moulton, Pennsylvania Hospital for the Insane, West Philadelphia, Pa.; William Osler, 1 West Franklin street, Baltimore, Md.; Charles W. Pilgrim, Hudson River State Hospital, Poughkeepsie, N. Y.; Theophilus O. Powell, Georgia State Sanitarium, Milledgeville, Ga.

